# Patterns, Entropy, and Predictability of Human Mobility and Life

**DOI:** 10.1371/journal.pone.0051353

**Published:** 2012-12-26

**Authors:** Shao-Meng Qin, Hannu Verkasalo, Mikael Mohtaschemi, Tuomo Hartonen, Mikko Alava

**Affiliations:** 1 Aalto University, Department of Applied Physics, Espoo, Finland; 2 WR WIRELESS RESEARCH Ltd, Espoo, Finland; 3 Zokem Ltd, Espoo, Finland; Umeå University, Sweden

## Abstract

Cellular phones are now offering an ubiquitous means for scientists to observe life: how people act, move and respond to external influences. They can be utilized as measurement devices of individual persons and for groups of people of the social context and the related interactions. The picture of human life that emerges shows complexity, which is manifested in such data in properties of the spatiotemporal tracks of individuals. We extract from smartphone-based data for a set of persons important locations such as “home”, “work” and so forth over fixed length time-slots covering the days in the data-set (see also [1], [2]). This set of typical places is heavy-tailed, a power-law distribution with an exponent close to −1.7. To analyze the regularities and stochastic features present, the days are classified for each person into regular, personal patterns. To this are superimposed fluctuations for each day. This randomness is measured by “life” entropy, computed both before and after finding the clustering so as to subtract the contribution of a number of patterns. The main issue that we then address is how predictable individuals are in their mobility. The patterns and entropy are reflected in the predictability of the mobility of the life both individually and on average. We explore the simple approaches to guess the location from the typical behavior, and of exploiting the transition probabilities with time from location or activity A to B. The patterns allow an enhanced predictability, at least up to a few hours into the future from the current location. Such fixed habits are most clearly visible in the working-day length.

## Introduction

The digitalized world is now getting entangled with the daily life of almost everyone. This has various consequences from the trivial ones – “being always connected” - to less obvious ones. But, for the scientist this gives a chance to study human life in a quantitative fashion. Aspects that become available include the mobility (person X goes from A to B) statistics and the way a person reacts via a phone to some external action [3]–[5]. Quantitative sociology then becomes boosted and enhanced by the possibilities the phone-based data offers, and allows to study “motifs” and regularities in life [6]–[8]. A very important dimension brought by the omnipresent phone to the modern life is the social context and interactions it brings about. [9]–[14].

Human life can obviously be seen to be both random and regular, and its inherent complexity is manifested for instance in spatiotemporal tracks of individuals – “mobility”. We are in the following work concerned with three fundamental questions: how to describe the regularity of the patterns in the life of a person from day to day, what kind of variations there are around that, and how predictable are the features of mobility then after all? To this end, we exploit a novel data-set of smartphone users (Methods) which allows us to extract the locations the users are associated with and pick the most important ones. The raw data is first pruned and filtered. The goal is then to extract the significant locations for fixed length time-slots – “where was I from 8 to 9 in the morning?” [15]–[20]. The data is thus distilled into a discrete format: at time-slot T the user X is in location (or perhaps state) A forming thus triplets (X,T,A).

These places or locations contain the most obvious ones such as “home”, “work” and so forth, but it turns out that for both a typical person and the whole set the location statistics follow a fat-tailed distribution. The data in the form (X,T,A) can be with a clustering-analysis turned into sets of days, that is typical patterns (“working day” etc.): in a certain days the activity of X at T is A, while in others it is B for instance. A day at work does not completely follow the pattern of that particular person, so noise is superimposed on the pattern. A physicist would say that a person has “ground-states” of typical behaviors which are mixed with fluctuations.

A physics or information theory based measure for the fluctuations is the entropy, so that an individual person can be characterized by the number of patterns he/she can be “classified with”, and the personal life entropy. The entropy is useful to compare when computed before and after finding the patterns by clustering [21], [22]. We discover that much of the raw entropy comes from the fact that some individuals have quite a few regular patterns of life rather than from real randomness.

The next natural question is whether one can predict the location (or activity by indirect logic) of a person based on the current one. The simple probabilistic guess “I know what this person does typically at this time” works fairly well for some, but in general does not do well. This can be improved somewhat by considering separately all the sets of days (ground-states, again). However, the empirical transfer matrices (related to pairs of the triplets (X,T,A) and (X,T+1,B) from above) include more of the information in the same patterns of life: the predictability is much higher utilizing those and the further pattern-induced improvement is quite mild. The capability to predict mobility fairly well in fact extends quite a few hours into the future. This is somewhat surprising given that the predictability is for each person dependent on the personal entropy.

We also analyze as the most significant pattern the working days in the data-set, clustering the days to find such patterns (Methods). This is done from the same starting point of predictability, but with the idea of trying to gain understanding on the origins of the daily random deviations. In concrete terms, we find that for a majority of the users in the set the working day has a typical, personal length and a simple model suggests, that the daily changes to this can be understood as a combination of a psychological trend to stay longer or leave and the daily random reasons to shorten/prolong the stay at work. This means, that there is a strong degree of predictability again, and that it is influenced both by personal (or by a particular job-dependent) details and personal stochastic variations.

## Results

### Days of Phone Users and the Entropy

**Figure 1:**
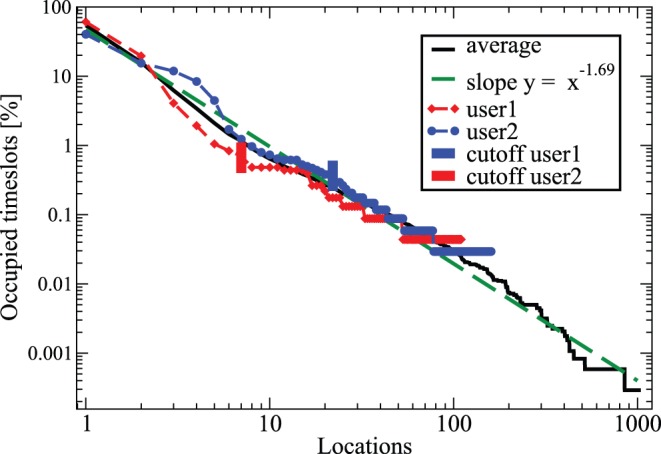
Important places in life. Average distribution (relative frequency) of locations in the timeslots. The data is averaged over all the different users in the set. A power-law fit to the statistics is also indicated, with an exponent −1.69. We also show two different random users with the “personal” statistics plus the cut-offs (see text) for the analysis of patterns.

The histogram of the relative time fractions that persons spend per location is shown in Fig. 1. The distribution appears to be wide, a power law with an exponent of about 1.7 (1.69 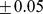); note that similar scale-free statistics surface in studies of human mobility [3], [23] though the two quantities are not the same. For each user in the further analysis below a threshold is used such that locations less frequent than 1% of the most common one are lumped together. Two individual users are demonstrated in the Figure 1 including the respective thresholds (7 and 22 significant locations). Note for instance how the statistics for user 2 are close to the averaged behavior. It is interesting that such regularities are found for individuals. The fraction of locations where some time is spent, that is how many locations are relevant, depends on the time during the day or the slot T (Supporting Information S1) [24].

**Figure 2:**
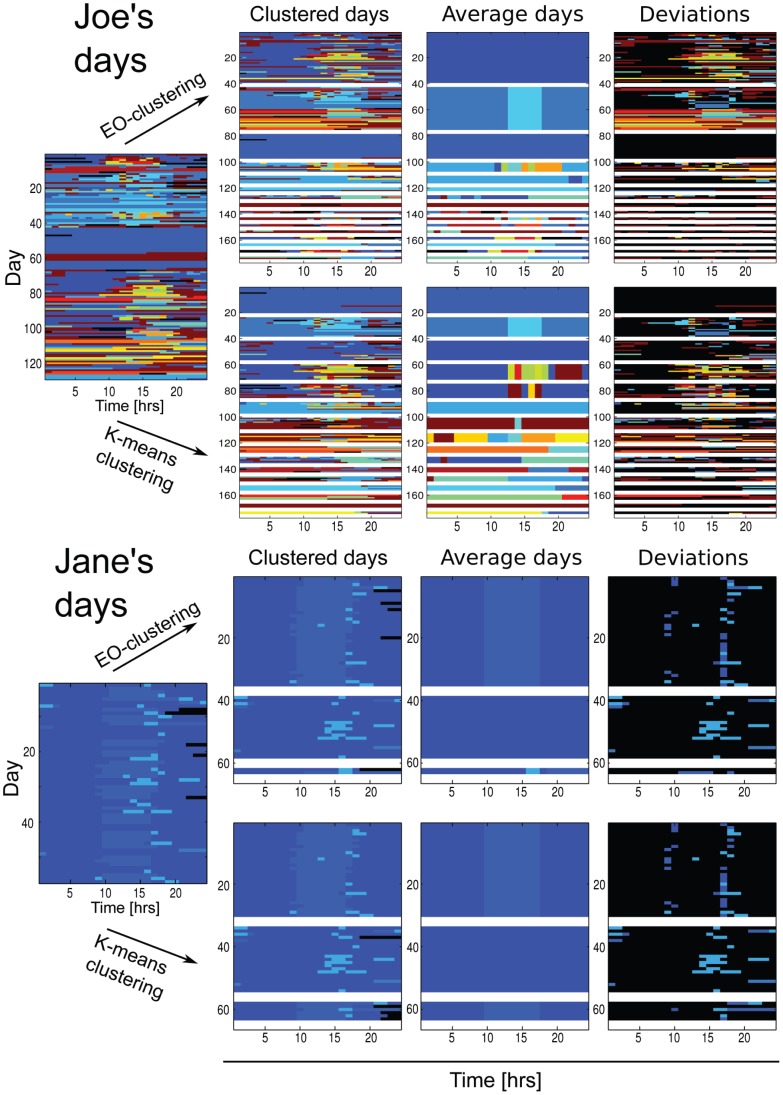
Patterns of life: Joe Random and Jane Regular. Upper panel: Clustering result for the *dayvector*s of *user Joe*. Lower panel: Clustering result for the *dayvector*s of *user Jane*. In both panels, in the first row are the results obtained with the extremal optimization algorithm and in the second row the corresponding result with the *k*-means clustering. Each color is one location – the clusters are separated by white lines. The first column shows the unclustered days. The second and third column show the clustered days and the corresponding average days of each cluster. The last column shows the deviations from the average day, where the color of the location is only shown if it deviates from the location of the average day of the cluster – otherwise the color is black.

Two example users, say Mr. Joe Random and Ms. Jane Regular, are depicted in Figure 2. The dominant patterns can be clearly identified as candidates for a typical “working day“ and a typical ”home day“. Two main features are the randomness or deviations present - consider a typical work day, where the noise is at both ends of the period at the work location - and the similarity of the clustering results. These two choices from extreme ends of the spectrum of the set of users illustrate the variety of behaviors. We also compare the results from two different clustering methods (EO and k-means, see Methods). The simple question here is how much the results from the analysis depend on the method applied.

Next, we compute a personal entropy over the time slots by
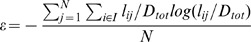
(1)where 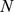 is the number of time slots in one day (columns), 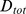 is the total number of days (rows), 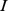 is the set of all visited locations and 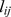 is the number of times location 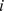 dominates time slot *j*. A low entropy means the user is regular and vice versa. However, part of the randomness is only apparent due to the patterns. The extreme limit is when the 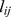 split perfectly among the different patterns (e.g. one is only at work in a certain slot in one, and elsewhere in the others). Thus one should consider a clustered entropy that gives in that limit the correct result, zero. Such one is the weighted average of the single cluster entropies:
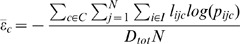
(2)where 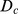 is the number of days that belong to cluster 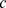 and 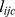 is the number of times location 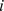 is the most dominant location at time slot 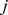 in cluster 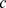, 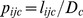 and 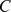 is the set of clusters. The drop in entropy due to the clustering is then defined as



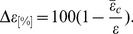
(3)This gives also a measure for the “goodness” of clusterings (the higher 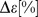 the better). When considering entropies one should recall that the clustering has been done a priori, without using entropy change to guide it. One possibility would be to look for the patterns that minimize the entropy, in other words. For the *k*-means case, the example which minimizes the distances to the cluster centroids is picked, which is in practice close to taking the smallest entropy value over the trials.

**Figure 3:**
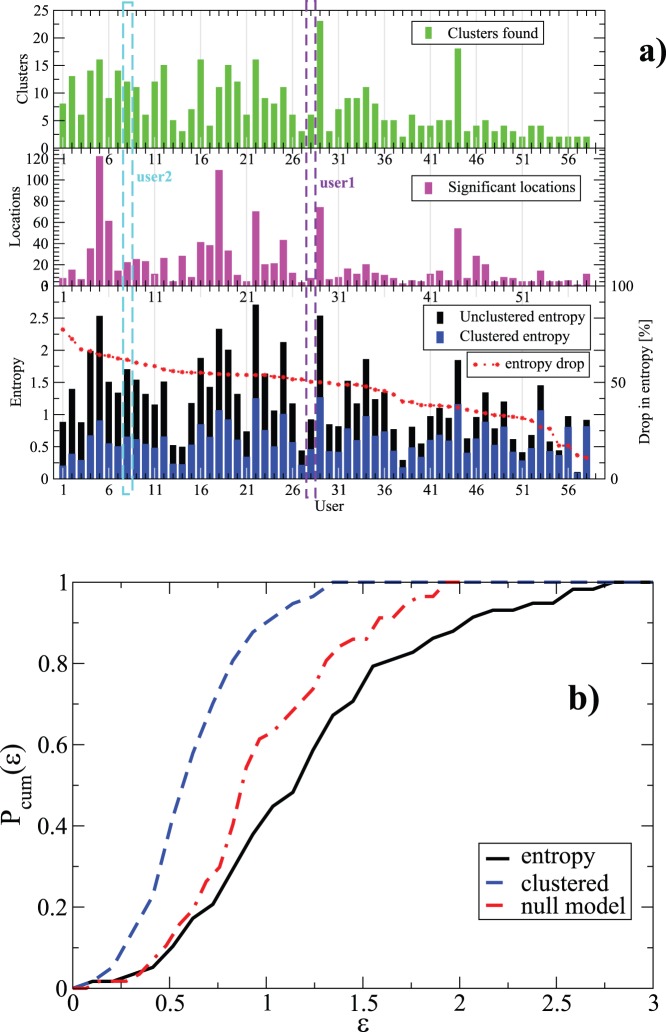
Statistics of patterns, and life entropy. a) The clustering results for 58 accepted users with the first place definition (off-line clustering) and a time slot of 1 hour. The clustering was done with the EO algorithm and 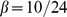. b) The cumulative distributions of the entropies for the three cases - bare entropy, reference model, and after clustering.

Joe and Jane exhibit different entropies and relevant patterns. Joe has a bare entropy of 2.70 decreasing to 1.25 and 0.78 after EO and *k*-means clusterings, respectively (54 and 71% changes). Joe’s life has 16 typical patterns. For Jane (3 patterns) the entropy is 0.44, and after clustering 0.21 (53% decrease). The results over the user set are summarized in Fig. 3a, ordered according to the entropy drop. A picture emerges of large entropy variations from user to user, and a trend in the entropy with the numbers of significant locations and patterns. The latter two fluctuate from person to person, but not with any kind of power-law statistics as that seen in Fig. 1. There are thus two contradicting observations: the entropies vary quite some, while the location statistics present regularities. The cumulative entropy histograms are presented in Figure 3b. It appears that they are quite close to *log-normal* distributions, though the tail properties can not be resolved convincingly with the histograms at hand. The question of where do the “rare” locations that exist in the location data end up or what they signify has an easy answer: a direct comparison of the noise superimposed on the patterns reveals that as one could expect the more rare locations are found (more frequently) in the noise (ie. when the actual location (X,T,A) does not agree with the pattern-indicated location (X,T,B), Supplementary Figure 26).

We used a null-model, included in the Figure 3b, to compare the entropies: assume the pattern(s) can be measured by a concentrated probability 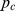 for a pattern (for the descriptive location), plus the residual variations around it in 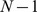 locations, where the 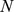 is found for each user separately as above. This means, that the 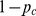 part is assumed to correspond to a completely random case. This would mean that the patterns (descriptive location) are just mixed with noise. Then, the maximal entropy can be written as 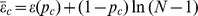. We find that the distributions are ranked so that the one for the patterned cases has the smallest entropies. This means most likely that additional information is hidden in the noise. The entropies for the user set do not extend in this case to very high values. The median entropies are 1.16 and 0.56, respectively. Such values have the simple interpretation that there are around three relevant locations for any slot, which decreases to less than two for a typical pattern.

### Predictability of Locations

To which degree one can predict a single user’s behavior from the current state, ie. location A at time T? If the entropy with patterns would be zero, this could be easy: just know the “actual pattern” to get a perfect accuracy. Such regularities or high predictability are analogous to what is indicated in some cases by common sense (“at sleep during night”, Supplementary Figure 27). We consider next predictions similar to weather forecasting: first, using the “average” behavior as in what is the expectation for a slot, and second, by asking what is the probability 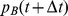 of the location 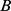 at 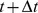 if the user was at 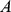 at 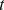
? The second method amounts to using the measured transition matrices and checking what they imply for the future from the current location, comparable to the recent Markov chain idea [25]. Since in both cases we can also restrict the days to one pattern at a time and average then over the patterns, we have in effect four different methods. The recent activities on “next location prediction” and similar topics quote a rather large number of related methods; our results below are better than any of those known to us from the literature [26]–[32] though Gambs et al. [25] reach comparable levels using GPS-based data. Etter et al. [32] for instance note also the possible roles of data sparsity, noise, and personal variations. Of these we analyze the two last ones below in more detail.

**Figure 4:**
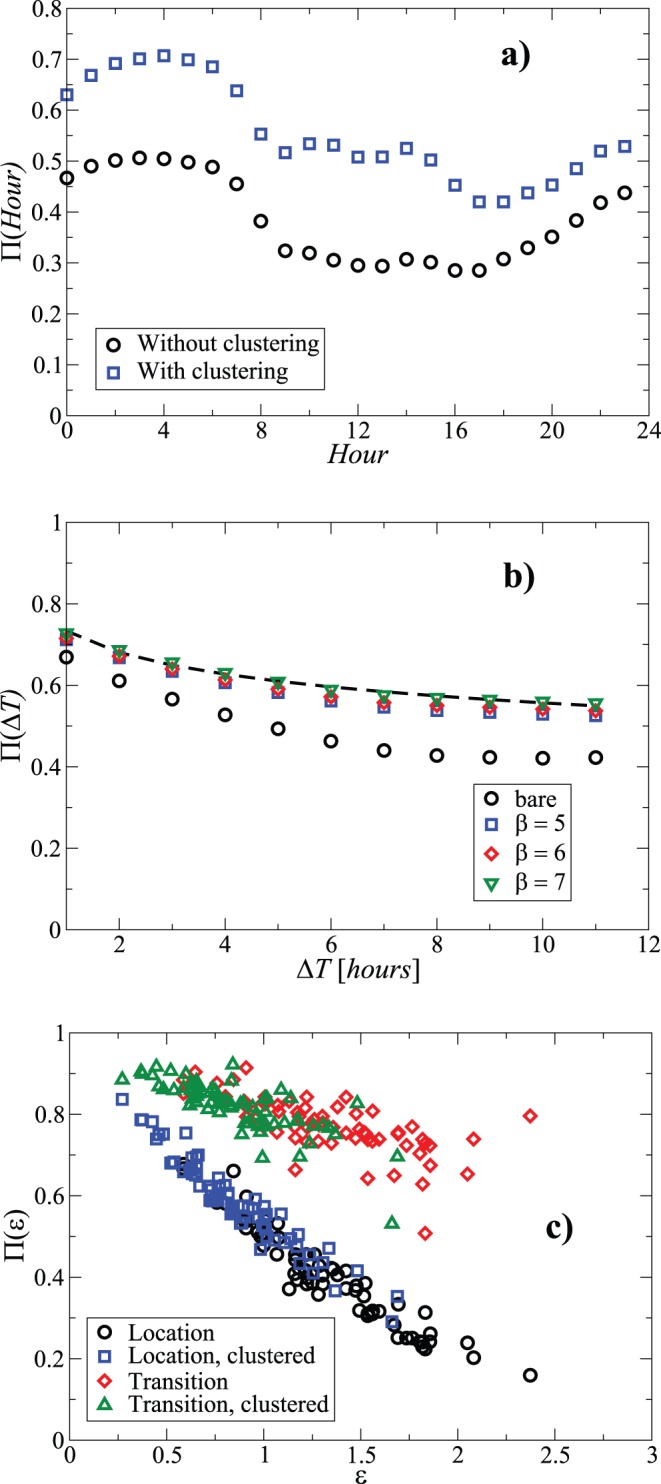
Predicting life. a) The prediction accuracy of the first, location based method in the case of with clustering and without as a function of time during the day. The clustering is from the EO algorithm with 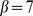. b) The predictions by the transfer matrix method for various 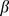 and 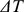 up to 12 hours. The dashed line is a logarithmic fit to the 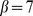 case. c) The correlation of the personal entropy and the resulting predictability for both the prediction methods (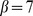). The entropy values are either the “bare” ones or those after clustering if that is used to aid prediction.

The first prediction quality is 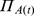, user will be at time 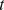. 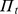 averaged over the whole day is the total prediction quality. With 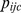 the probability to be at *i* at time slot *j* in pattern *c*, then, quite simply 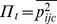, with the average over all *i*, *c*. This method amounts to utilizing the measured probabilities to get a a guess where a user would be - consider a two-state model where these locations would have probabilities 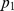 and 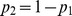, in which case such a guess works with the quality 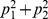. The results are shown in Fig. 4a. The prediction accuracy improves with patterns to 0.53±0.12 from 0.36±0.11 (without).

With the second method, considering the equivalent case by setting 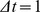 the accuracy is much higher. The average value changes adding the patterns from 0.74±0.07 to 0.78±0.06 (Fig. 4b), the best result of the four different methods tried. The small difference or change from the patterns implies that they are already quite well encoded in the original transition probabilities. This is natural since here we consider the transitions (or mobility), not the static probabilities. Thus in the case of the previous example one could find that the two states always change to the other at the next timestep: this has a quality of one. The maximum mean predictability is suggested by the method and data to be close to 0.8 even with further refinement in finding patterns, due to this small improvement. The upper limit of the last case, with patterns, as observed from the distribution of personal predictability values found here, is quite close to the maximum limit postulated for the predictability of mobility patterns, 0.93 [4]. This might not be surprising, that the predicability of daily routine is constrained by the randomness-induced limitations in mobility prediction.

The patterns imply that the transitions contain useful information, which then brings us to look at long-range predictability, 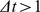. The quality decays logarithmically with 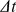, Fig. 4b, or possibly as a power law with a small exponent (Supplementary Figure 28). We have not considered longer-range predictions than those depicted here mostly due to data limitations. Surely this question is interesting and has non-trivial features - the sleep-wake cycle, the weekly cycle of work and leisure among others. The slow decay observed or measured implies that life in its regularity is - again given that there are such patterns - actually relatively quite predictable. In particular, note that the quality remains better than the average of the location method for 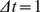, even up to time-delays of several hours. Below, we discuss the regularities of working days, where a high level of predictability is often quite given a priori.

Predictability has large personal variations (Fig. 4c) due to entropy if one tries predicting without exploiting the patterns. The range in which the first type prediction quality varies shows a substantial variation from “easy to predict” to “difficult to predict”, which correlates as stated with the entropy (Supplementary Figures 29–30). Joe’s life would be appear to be quite impossible to capture without patterns, but including those increases the predictability substantially “upto” the level in the case of Jane. In other words, the implication would be that we are slaves of our daily-life habits, but those can be quite plentiful and different and still exhibit typical deviations.

### Departing from Work

**Figure 5:**
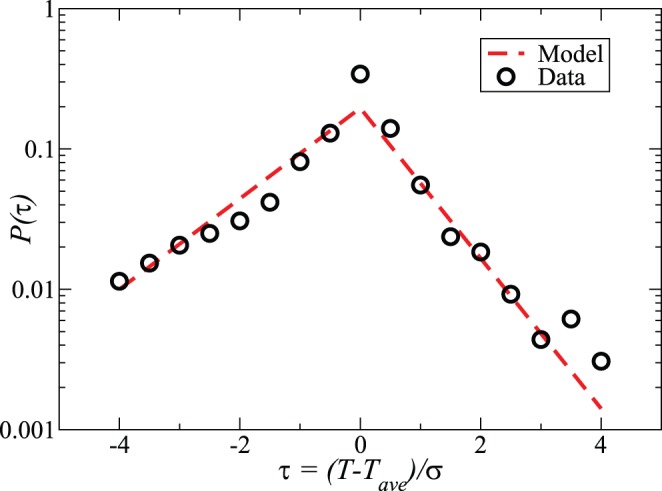
Predicting leaving work. Length-of-working-day distribution in scaled units and the model fit. The parameters for the leaving early and late -processes are 0.045 and 0.046, respectively (in inverse 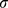-slot lengths).

The regularity in the data includes and implies long-range daily correlations: the behavior depends on the past. The easiest example of this is the working day length, which we consider next. Figure 5 depicts the distribution for the probability to leave from work (to any other location), for users that have regular working patterns and enough data, slightly more than a half of the total (Supplementary Figure 31). The data has been first scaled by using the mean and the standard deviation for all such users so that one is able to consider the sum distribution.

The tails of the likelihood are different on both sides of the maximum. The Figure 5 includes a fit of a model (see also Supplementary Figure 32). First we assume that the time spent at work is shorter in a particular day when one leaves for a specific reason (visiting a doctor, meeting friends/family…). The action of leaving from work has a likelihood per time step, which increases by a constant factor by each step until the day has reached its typical length. This assumption means that it becomes more easy to quit working if the resulting deviation from the usual is shorter. Sometimes on the other hand work requires extra attention. In the model, we assume simply that if the day lasts beyond the typical length the probability per time step to leave increases with another parameter. This means that it becomes more difficult to stay for each further step, a trend which is exactly the same as assumed for the other case of leaving early.

These assumptions boil down to two exponentially increasing trends to leave early or not to stay any longer, which can be used to fit a distribution to the data. For the data at hand, the model works fairly. The central assumption behind the model is that the average working day length has for each user a deterministic influence on the decisions to change once from the usual habit, perhaps a triviality in many professions. This conditions the patterns over a large time span, as seen earlier in the context of long-range mobility prediction (compare with. Fig. 4b). We also tried to correlate the departure from work with the personal communication records. This amounts to looking at call and text message patterns and partners (numbers for in- and out-coming communications considered also in the light of their frequency). The idea would be roughly: “I go since I made a call or got a call”, possibly also conditioned on the quality of the communication: either a rare or frequent contact as deduced from the randomized phone number. However, such attempts were left inconclusive. One reason is that the persons considered in this study on average communicate (calls, text messages) fairly rarely, so any possible causality can not be derived from data. The lack of correlation in any case implies a relatively minor role of such communications in enforcing a particular departure from routine.

### Conclusions

To summarize, smartphone -based studies allow for quantitative insights into the fundamentals of human life. We have here analyzed such data with the approach of first extracting patterns and a measure of randomness, and then analyzing the predictability of individual mobility. This utilizes the coverage in space and time available from the data-set. All in all, this means that such spatiotemporal patterns, together with the personal randomness, define quantitatively individuals and populations. Patterns and entropy relate to the degree the daily locations or activities are predictable. We have demonstrated a fairly good quality (0.78 in the best of the four methods tried).

The data-set we have at our disposal is just sufficient for the present purposes. The user panel, with its particular socio-economic and geographic background (for a comparison, consider Ref. [33]) and its size may introduce various bias, which however pales in importance if put into contrast with a few bigger issues in the context of human life analysis. We already find noticeable variations in the simplest personal quantitative characteristics: the number of locations to describe the personal life, the number of patterns, and the entropy, in the case at hand. It appears that the consequent realistic predictability arises from the presence of both patterns and noise in the life. Quite good predictability - the best such to our understanding so far - is found from the combination of using mobility and the discovered life patterns.

One follow-up question is: how do entropy and predictability change from culture to another, including on-line games and so forth [34], or during longer spans of an individual’s life. We are here looking at both of these quantitative characteristics with the somewhat contradictory assumption that the persons described by the data are “in an equilibrium”. Thus, another important question is: how does the eventual lack of stationarity matter here, and how should it look like in the data? Life is not periodic, as it might be in weekly or monthly patterns over a finite time span, and moreover a society or culture is in a state of permanent flux. What do the inevitable trends and transients look like, for a person? Are there collective phenomena [35] quantified by the entropy, patterns, and predictability computed over long but finite windows in time - as during the times of a crisis in a society? Such questions will need to be answered by access to much larger data-sets.

## Methods

The location data consists of the raw cell ID timestamps and the coordinates of the corresponding base-stations from a smartphone-based application [6], [36]–[38]. The mapping of cell ID is difficult for issues inherent to the mobile phone technology [39], for which reason we used two methods (Supporting Information S1, Supplementary Figures 1–3): offline clustering [15] and a fingerprint based method. This kind of comparisons are useful since such approaches can not be perfect. The original data set has more than 500 users, from a commercially conducted “user panel” in southern Finland during 2008–10, before processing. The participants (“panel members”) gave written consent (“user agreement”) also concerning the gathering of the data and its use for various purposes in the anonymized format, including scientific studies. The location data is further coarse-grained into time-slots of uniform length resulting in the triplets (X,T,A) from above (see also [40], [41]). Here, we use the simplest possible approach where the location A for the slot is chosen by a majority rule from the detailed location information during the slot length (see in general the Supplementary Figures 4–11).

The data contains gaps, typically since the phone is closed down and so is then the data gathering application (Supplementary Figures 12–14). The time-slots missing are partly covered by a padding technique (user X closes the phone at home from 01.00 to 05.00 being a typical case). Such gaps seem in general to contain some information on the user behavior. Finally, days with more than 30% of missing data after this procedure were entirely excluded, reducing the missing data from initially almost 50% to less than 1%. The slot length has been varied from 30 minutes to 2 hours, out of which we show in this work representative results for the 1 hour case (recall the daily 24 hour cycle). The original data-set of several hundreds of users is at the end pruned upto 66 persons, for each of which at least 30 full days of data suitable for analysis were found. In addition to the location data, we also have access to a call and message data set for the same persons - except for the “padded” time slots of course. This allows in principle to study the correlations of user activity with both the communications patterns and individual communication events such as text messages and calls, including the anynomized phone numbers.

The patterns are resolved by a cluster-analysis (Supplementary Figures 15–26). The analysis is done to establish the typical days or a set of characteristic behaviors. Note that we assume directly that the human behavior here can be classified in this way : that there is not simply a continuous spectrum of eigenbehaviors in contrast to a discrete set [6], and that it is reasonable to consider this on the particular level of time-discretization (1 hour slot length). The easiest division here might be “work-days” and “weekend-days - that separate to subclasses as “Saturday” and “Sunday” - but a priori this is not given.

We use two methods to compare the influence of the technique; naturally the result depends e.g. on the similarity metric used for a pair of day-vectors. The first is 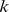-means clustering and Euclidean metrics on a binary expansion (see Ref. [6]) of the data-set. The second method is community detection on weighted graphs, where the days constitute the vertices and the weights depend on the weighted Hamming distance 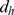 between any two as
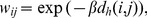
(4)where 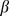 is a parameter. We apply an extremal optimization method (EO) [42], so that we can find the number of clusters 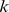 as a self-organized outcome. We compute a single clustering, while for the 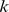-means we do 200 runs with random initial conditions.

## Supporting Information

Supporting Information S1 **Information and further details on the data handling and processing, and results (6 MBytes).**
(PDF)
