# Supplementary material for: Patterns, Entropy, and Predictability of Human Mobility and Life
Source: PLoS One. 2012 Dec 26;7(12):e51353. doi: 10.1371/journal.pone.0051353 (PMC3530566; doi:10.1371/journal.pone.0051353)
Supplement: Supporting Information S1 — Information and further details on the data handling and processing, and results (6 MBytes). (PDF) [file pone.0051353.s001.pdf]

Patterns, entropy and predictability in human life
S. Qin, M. Mohtaschemi, T. Hartonen, H. Verkasalo, and M.J. Alava: Supplementary information

## Contents


**1 Data and Methods** **1**
- 1.1 The dataset . . . . . 1
- 1.2 Location analysis . . . . . 2
  - 1.2.1 First approach . . . . . 3
  - 1.2.2 Second approach . . . . . 5
  - 1.2.3 Creating *dayvectors* . . . . . 6
- 1.3 Data properties . . . . . 8
  - 1.3.1 Available location data . . . . . 8
  - 1.3.2 Gaps in the location data . . . . . 12

**2 Supplementary discussion** **16**
- 2.1 Clustering results . . . . . 16
  - 2.1.1 Entropy after clustering . . . . . 17
- 2.2 Predictability . . . . . 23
  - 2.2.1 Method 1: The expectation of daily life. . . . . . 24
  - 2.2.2 Method 2: Transition matrix . . . . . 24
- 2.3 Voice call and Message data: Work-Home cycle . . . . . 27


# 1 Data and Methods

## 1.1 The dataset

The data used is in this work originates from a reality mining panel collected in Finland during 2010. In total more than 500 persons participated with their mobile phones. However, as became clear during the processing of the data, not all users participated long enough to provide useful data for the analysis by methods explained in this work. In order to participate, a user had to install a special monitoring software on his/her phone. This software was designed to collect a broad amount of information about a users mobile phone usage. Among these was also the GSM base-station the mobile is connected to (for basics of GSM network see [1]). Other information that was collected included for example log information of phones and messages, bluetooth encounters and calendar information. The software sent the gathered data on a regular basis to a data collection server.

The location part of the dataset, the base-station timestamps, were collected in the following manner: The current base-station for a mobile was logged after each handover — in addition to this, all except the very first versions of the

reality mining software logged the current base-station at least once every 30 minutes. A few random lines of the raw location data are shown below.

```
User ID   ISO-timestamp         Basestation ID
95xxx     2010-xx-xx 21:08:35   1398
95xxx     2010-xx-xx 21:09:26   1400
95xxx     2010-xx-xx 21:09:44   1398
95xxx     2010-xx-xx 21:18:43   7350
...
```

The dataset contained also the coordinates of all occurred base-stations. Thus a users location can be estimated as the location of the base-station the users mobile is currently connected to (cell ID positioning see e.g. [2]). The other available data was provided in a similar format with the user ID, time stamp and the actual data entry in one flat file for each type of data collected.

The dataset was anonymized in such a way that participants were distinguished only by numerical identifiers (user ID). Real phone numbers of the participants and the like were not available to the researchers. In addition to this, during the whole scope of the study, privacy issues were a major concern and the publishing of any material that could be used to identify any individual participants (e.g. detailed location records) has been avoided.

## 1.2 Location analysis

It was noticed that while being most of the time plausible, there were some clear errors in the raw location data. These included for example jumps over several hundreds of kilometers in a few seconds and a jump back to the origin within another few seconds. There are several sources where these errors might come from, for example: wrong timestamps, wrong mappings between tower ID and location or a disturbed connection to the data server. Before proceeding any further, these errors were filtered out.

The data cleaning was done in the following way: If the distance of two successive locations (base stations) was longer than 100 km and the time interval less than 30 minutes (corresponds to a speed of at least 200 km/h) the later location is removed. All directly following locations with the same base-station where removed as well. Also all locations with wrong timestamps not within the actual measurement period were removed. These were caused due to wrong time settings in a users mobile for example due to a hard reset. The procedure as explained above proved to work well in removing the clear errors from the raw position data.

Due to issues like repeated handovers between neighboring cells (cell jitter see [3]), the raw cell id location information does not work well for the purposes of this work. As it turned out there has been already quite a lot research about the place definition problem. We are now using two different algorithms.

In order to make good predictions on the behaviour the definition of places should be as accurate as possible. In a bad place definition for example *HOME* (assuming here that the person under consideration has only one home) could be split into two separate positions. A move from *HOME1* to *HOME2* would not be of much sense and would forge the movement pattern of this particular user.

The first algorithm (Fig. 1) is a slightly adjusted version of Laasonens offline algorithm [3]. This algorithm uses the initial base station data and places are defined as clusters of base stations. The algorithm basically tries to detect

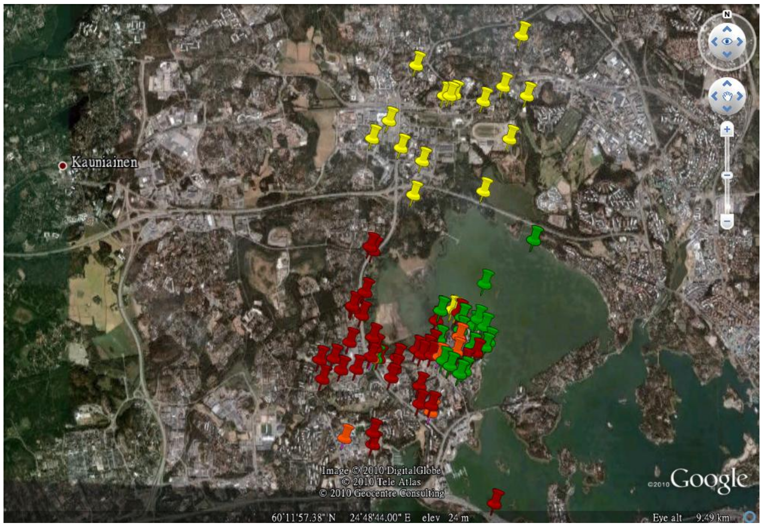


**Supplementary Figure** 1: A few important places detected by the base station approach (def1). A place is defined as a set of base stations. The drawing pins present the positions of base stations. Each color is one location.

clusters of base stations in which there are many oscillations. A problem with this method that the initial clusters might overlap. However the places might become too large, if one simply merges all overlapping clusters. A slightly modified version (online version) of this algorithm is able to run on a users cell phone without knowledge of the locations of the base stations.

### 1.2.1 First approach

Fingerprint based methods (see e.g. [6] [3, 4, 5]) work well for the location data at hand. In this work Algorithm 1, which is the off-line clustering algorithm by Kari Laasonen [3] with two slight modifications, is used for obtaining individual places. Basically this algorithm goes through the sequence of base-stations and groups the base-stations with frequent oscillations between them into clusters — these clusters are the extracted places. The reasoning behind this is that the mobile of a user staying at one place, will connect to one of the closest base-stations and occasionally switch to one of the other closest base-stations. It is the set of these closest base-stations that is searched for. In Algorithm 1 $S$ is the sequence of base-stations that is gone through, $R$ is the cluster of base-stations that is currently tested, $T$ is the set of tested clusters ($T \subset \mathcal{P}(S)$) and $P$ is the set of accepted clusters ($P \subset T$). $\gamma(R)$ is the ratio function, a high value of $\gamma(R)$ indicates frequent oscillations between the cells in $R$ — the notation is the same as in the original paper [3], where the algorithm is explained in more detail. The modifications are discussed below.

The first modification is that an additional condition has been added which forbids the clusters to grow geographically too large (line 8 in Algorithm 1). This can be done since the position information for each base-station is known in our case. In particular the condition states that the distance between any two base-stations in the same cluster can not be greater than 3 km. The purpose of this rule was to neglect clusters too large to represent any single meaningful places. Test runs verified that the modification improved the results.

The second change is that the maximality of $r$, which holds the value of the ratio function $\gamma(R)$ of the corresponding cluster, is not tested explicitly on all subsets of $R$ — as it is the case in the original definition. Instead $r$ is only compared to the highest value of the same iteration over $k$ (line 13 and 14 in Algorithm 1). This change was done since the comparison of all subsets was computationally too expensive in the implementation. As a side effect additional clusters are added to $P$. These clusters and possible overlaps are handled separately from the initial algorithm: First $P$ is sorted by the values

**Algorithm 1** The used algorithm for detecting meaningful places

1: $k \leftarrow 1$
2: **for** $i \leftarrow 2$ **to** $|S|$ **do**
3:   $R \leftarrow \{s_i\}$
4:   **for** $j \leftarrow i - 1$ **downto** $k$ **do**
5:     $R \leftarrow R \bigcup \{s_j\}$
6:     **if** $R \notin T$ **then**
7:       $T \leftarrow T \bigcup \{R\}$
8:       **if** $\mathrm{diam}(G_R) > 2$ **or** $\max\{\mathrm{d}(s_{i1}, s_{i2}) > 3 \text{ km} \mid s_{i1}, s_{i2} \in R\}$ **then**
9:         $k \leftarrow j + 1$
10:         **break**
11:       **end if**
12:       $r \leftarrow \gamma(R)$
13:       $r' \leftarrow \max\{r, r'\}$
14:       **if** $r > 1$ **and** $r \geq r^{'}$ **then**
15:         $P = P \bigcup \{R\}$
16:       **end if**
17:     **end if**
18:   **end for**
19: **end for**

of $r$ thus that the first element in $P$ has the highest $r$. In order to get rid of the clusters that are not minimal in the sense of the original definition, the Algorithm 2 is used. In this algorithm on line 4: if $i$ is a subset of $j$, then $j$ is not minimal and is therefore removed from $P$. If $j$ is a subset of $i$ then $j$ is also removed, since $i$ has a higher $r$.

**Algorithm 2** Cleanup step of the initial algorithm

1: **sort** $P$ by $r$
2: **for** $i \in P$ **do**
3:   **for** $j \leftarrow i + 1 \in P$ **do**
4:     **if** $i \subset j \bigvee j \subset i$ **then**
5:       $P \leftarrow P \setminus j$
6:     **end if**
7:   **end for**
8: **end for**

Many of the clusters in $P$ might conflict with each other. There are several possibilities on how to handle these, off which the simplest is to merge overlapping clusters. The approach here is to merge overlapping clusters in $P$, if the intersection is greater than by two base-stations (elements). By doing so smaller clusters are achieved which should provide better results. On the other

hand, due to the remaining overlaps, it is not possible to define an unambiguous mapping from the base-station information to the place information. This does not constitute much of a problem in the further analysis.

The final step in getting the possible places at a given time $t_1$ is as follows. Assume that at time $t_1$ the mobile phone is connected to cell $c$. The set of possible places is the set of clusters in $P$ the cell $c$ belongs to. If $c$ does however not belong to any cluster then the place is $c$ itself. If there is no cluster then $c$ has no neighboring cells between which significant oscillations would occur i.e. the connection between the mobile and $c$ is assumed to be stable. This could for example be the case in an open countryside where there is only one base-station in reach.

The place detection in [3] continues by extracting from the set of places the meaningful ones — the bases. This step is here however omitted in favor of a fixed timeslot definition which will be discussed later on.

### 1.2.2 Second approach

The second approach (Fig. 2) uses the location data which has first been prehandled in order to maximize the accuracy after we clean the data set. If we want to know the position of a user at time $t$, the position of four base-station timestamps around $t$ are needed $\mathbf{B}(t_1)$, $\mathbf{B}(t_2)$, $\mathbf{B}(t_3)$ and $\mathbf{B}(t_4)$, and $t_1 < t_2 < t < t_3 < t_4$.

$$
\begin{aligned}
a(t) = \frac{1}{4}\Big\{ \quad & \mathbf{B}(t_2) + (\mathbf{B}(t_3) - \mathbf{B}(t_2))\tfrac{t-t_2}{t_3-t_2} \\
+ \quad & \mathbf{B}(t_1) + (\mathbf{B}(t_4) - \mathbf{B}(t_1))\tfrac{t-t_1}{t_4-t_1} \\
+ \quad & \mathbf{B}(t_2) + (\mathbf{B}(t_4) - \mathbf{B}(t_2))\tfrac{t-t_2}{t_4-t_2} \\
+ \quad & \mathbf{B}(t_1) + (\mathbf{B}(t_3) - \mathbf{B}(t_1))\tfrac{t-t_1}{t_3-t_1}\Big\}.
\end{aligned} \tag{1}
$$

We divide the globe (or the part of the globe a user has visited) into a square grid with 0.002 in latitude and 0.002 in longitude. So the size of each grid depends on its latitude. We locate user's location every 15 mins. Therefore the accuracy of the linger time for all cells is also 15 mins. The meaningful place in this definition should be a continuous area on the map where the linger time of its cells are similar. We use three steps to recognize these meaningful places in this method,

(1) We need to move some unimportant places where user visits casually. If the linger time of a cell is less than 45 minutes, one can believe the linger time of this cell is 0. We use a very small threshold 45 mins, considering that for some places, like the gym, the user will not visit a lot but they are important meaningful place.

After we use a very small threshold in step (1), we find some meaningful places like "office" and "home" are always connected by the road. We must recognize the "office" and "home" respectively. Fortunately, we find the linger time of "office" and "home" are different a lot with "road". Then we can repeat the following steps to define meaningful places.

(2) Find the largest linger time cell as the center cell in all cells which do not belong to any meaningful place.

(3) Define a continuous area around the center cell as a meaningful place where the linger time in these cells is longer than the 1% of the center cell.

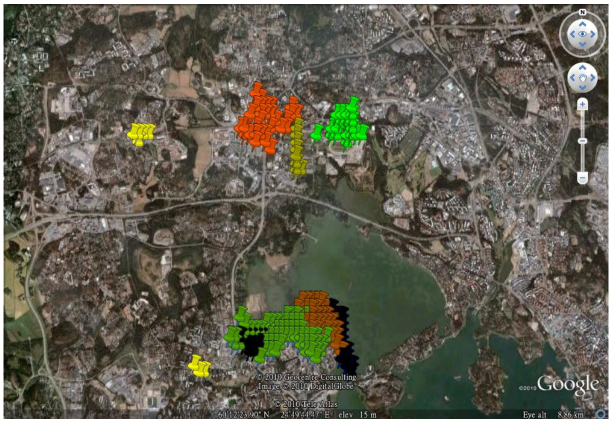


**Supplementary Figure** 2: Some important places of user 1, detected by grid approach. Drawing pin presents the center of the grid. Each meaningful place is a continuous region where the drawing pins have the same color.

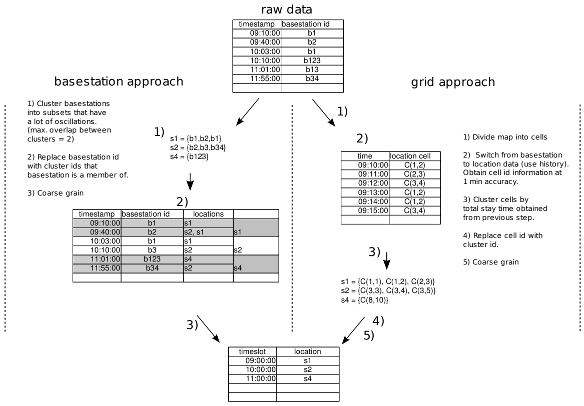


**Supplementary Figure** 3: Schematic presentation of the process from raw base station data to *dayvectors* for place definition one and two.

For each user, we repeat step (2) and (3) 50 times or until we can not define more places. In other words, each user has 50 meaningful places in the most. The benefit of the location grid based approach is that it can be directly extended to any kind of localization technology (gps ...).

### 1.2.3 Creating *dayvectors*

Independent of which place detection algorithms were used, one can now create for each user and each day a vector which contains the visited locations. This is just what Eagle and Pentland [7] have done in the special case of a "three-state" system (work/school, home, elsewhere). The wanted time resolution is determined by the used time window. With a time resolution of 12 hours each vector would have two entries. The process from raw base station data to *dayvectors* for both place definitions is visualized in Fig. 3

We define the location of one timeslot to be the one where the most time has been spent during that timeslot. Another option would be to use the location

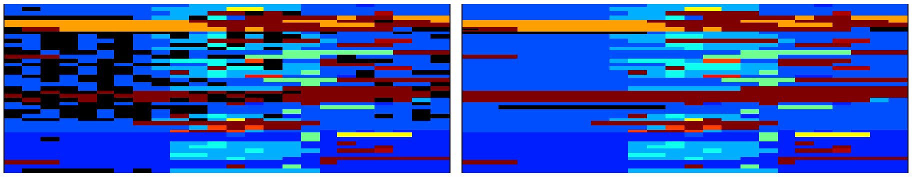

(a) raw (b) padded

**Supplementary Figure** 4: A few *dayvectors* of a user at a time window of 1 hour (a) before (b) after handling missing data. Each row represents the locations of one day. Each color is a different location. Slots with no data are black.

Table 1: Set of rules by which the missing data is padded.

| Name | Criteria | Example | Max duration |
|---|---|---|---|
| Gap1 | Begin = End place | AXXXXA | 8 h |
| Gap2 | Begin ≠ End place | AXXXXB | 4 h |

with the longest continuous stay time in the timeslot. However, it is likely that the differences of these two definitions are rather small. A benefit of using time slots is that one can handle overlapping locations without problems.

There are several reasons which can result in missing data. The largest gaps that occurred in the dataset, which are of the order of several days, are most likely due to the fact that a user has temporary removed the reality mining application from his/her phone (type $A$). Another source is due to the earlier versions of the reality mining application itself, which sent location data only when a cell transition has occurred (type $B$). There might be long time periods without cell transitions ergo missing location data. Most of the missing data during a single day is thought to be of the this second type. The third reason for gaps is that a phone is not connected to any base-station (type $C$). This is the case when the phone is either switched off (by purpose or accidentally e.g. due to an empty battery) or the phone is out of the reach of the mobile phone network. Missing data does cause problems in the following data analysis and thus it is tried to pad with reasonable estimates for the location where ever possible.

In the current approach the missing data is handled as follows: If the most dominant place in the timeslot before and after the gap is the same and the gap length is less than 8 hours, then the gap is filled up with the place at its edges. If the place before and after the gap are not the same, then the gap will be filled up with the place before the gap, if the gap-length is less than 4 hours. This set of rules is shown in a more comprehensive way in Table 1. The procedure results in accurate place data, if the missing gaps are of type $B$. If the gaps are due to some other reason (type $A$ or $C$), then there is no guarantee that the result is correct. It might be even conceivable that a user switches off his/her phone with the particular purpose of hiding his/her place.

Since the operation is done on the coarse-grained data, the procedure is independent from the used place detection algorithm and the format of place data, but on the other hand different gaps might be filled up if the time-resolution is changed (8h versus 4h fill-up if start and end place are the same). The effect of

Table 2: Criteria for including a day and a user into the analysis.

| Name | Criteria |
|---|---|
| Accept day | $<30\%$ empty timeslots |
| Accept user | $>30$ days accepted |

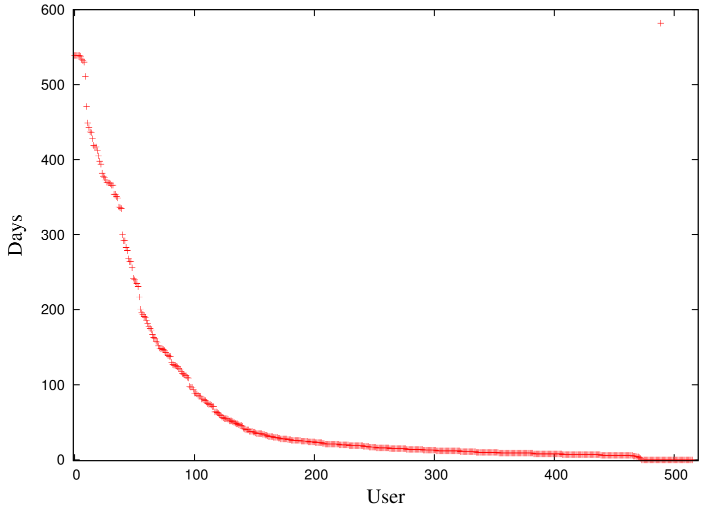


**Supplementary Figure** 5: Available days for all participants of the user panel prior to any data handling.

the missing data handling is demonstrated in Fig. 4b.

Not all gaps are filled by the method as described above. The parts of the dataset that were not complete enough were rejected. In particular, days with more than 30% of empty time slots after the fill-up procedure were not accepted. In addition to this, a user was excluded from the further analysis if there were less than 30 days of accepted days (see Table 2).

## 1.3 Data properties

### 1.3.1 Available location data

Only around one out of ten users (depending on the used place definition) had usable location data for more than 30 days. The raw days for all users are shown in Fig. 5.

The accepted days for the first place definition are shown in Fig. 6 a) and for the second place definition in Fig. 6 b). The accepted days for the two methods are compared in Fig. 7 — the two methods give slightly different results. With the second method a few more days are accepted on average. However in both cases around 60 users had over 30 days with enough location data.

What do the typical dayvectors contain on average? The distribution of the locations in the timeslots are shown in Fig. 1 of the main article for the first place definition and in Fig. 8 for the second.

As can be observed from Fig. 1 of the main article, the total number of places with the first place definition might be rather large. Many places might occur in only one or just a few time slots. What is the actual number of crucial

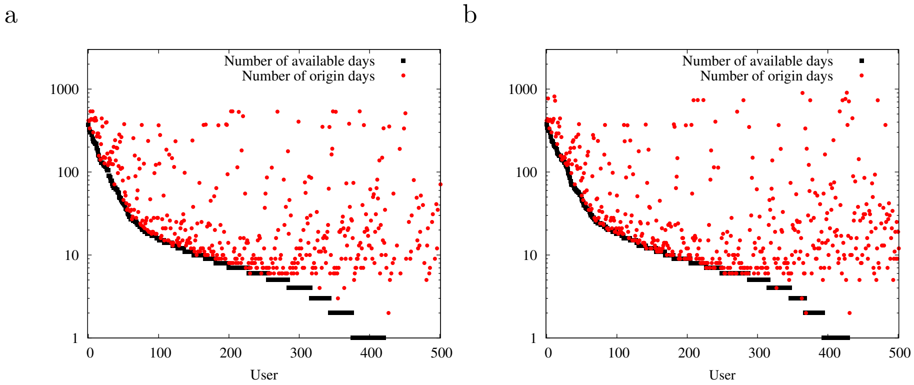


**Supplementary Figure** 6: Accepted days a) def1 and b) def2 for all users.

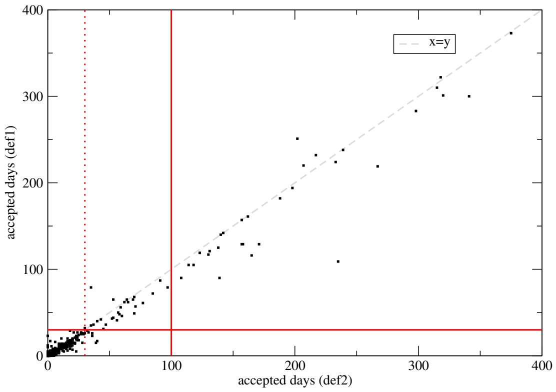


**Supplementary Figure** 7: A scatter plot of the accepted days for def1 versus accepted days for def2. Each point is the data for one user. The red lines mark the acceptance criterion for users in the analysis (30 days for def1, 100 days for def2).

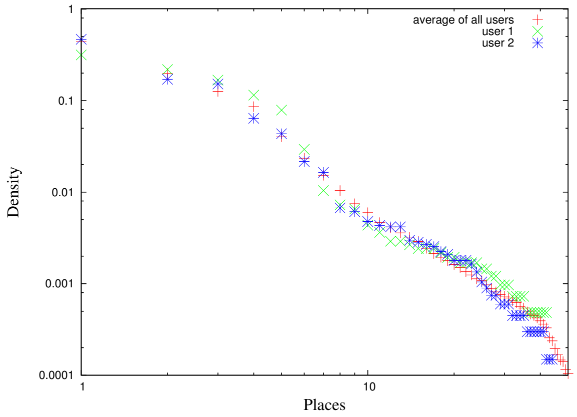


**Supplementary Figure** 8: Distribution of significant locations for the accepted users with place definition two. The results were obtained with a timeslot of one hour.

places that have to be tracked for a given user in order to be able to get the correct live patterns? Starting with a users coarse-grained place data for a given time window (say e.g. 1 h) the places are sorted by the number of times they occur in the *dayvectors*. The place $l_i$ is the one that is the $i$th frequent place, further let the $n_i$ be the number of times place $l_i$ occurs in the *dayvector*s. Relevant places are further defined as the ones with $n_i > 0.01\ n_1$ ( $n_1$ is likely to be home). The other places are merged into a single place *else*. Keeping more places did not really improve the results, as test runs have shown. One should notice that the used definition does also depend on the used timeslot — with a shorter timeslot the number of relevant1 places is probably larger. No significant relation between the number of available days and the number of locations found is evident for the first place definition (see Fig. 9).

The distribution of the number of important locations is shown in Fig. 10. An analysis of the functional form of the cumulative distribution reveals that it does not adhere to a simple form, say a power-law times an exponential cut-off ("$x^{-a} \times \exp -x/b$"). On the other hand the distribution is clearly not peaked and rather wide. We return to the significance of the empirical features when discussing entropy.

For the second place definition, we delete some cells with the linger time smaller than 45 mins. Then we also limit the max number of places for each user. From Fig. 8, we can see all users are far from close to this limit. In other words, we collect all meaningful places we can recognize in the second place definition. However, in some cases, we know where the user is but we cannot define the user's meaningful place. This is since the user is on the road to somewhere or at some unimportant place. Therefore, we define that a place means "others" to describe this situation.

To summarize with definitions 1 and 2 slightly different placevectors are achieved. Definition 2 uses the location data directly and is thus probably more accurate.

The fraction of actual locations $f_i$ is shown in Fig.11 in the case of 30 minute slots. The fraction is studied as a function of the time of day. $f_i$ is the average of relative fractions over the dataset (ie. the ratio of locations found at time-slot $i$ for user $u$ over $s_u$, the number of significant locations for user $u$). The number

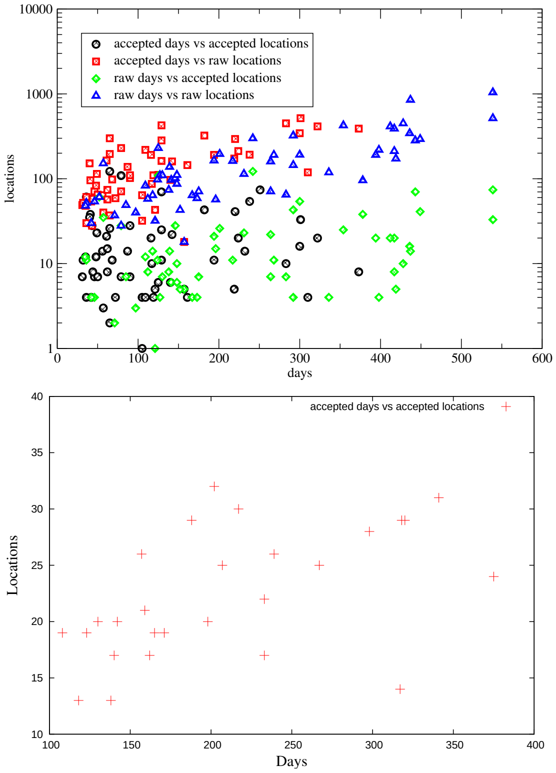


**Supplementary Figure** 9: Days found versus locations found with the first place definition (upper) and second definition (lower panel).

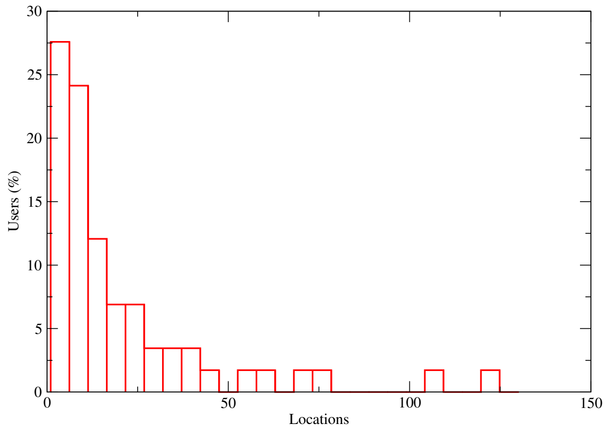


**Supplementary Figure** 10: Distribution of important locations found with place definition one.

of visited locations should be a function of the time of the day; It is intuitively clear that, generally speaking, people tend to stay mostly at home during the night and most of the activities requiring a switch in location occur during day time. This trend can be observed in the Figure.

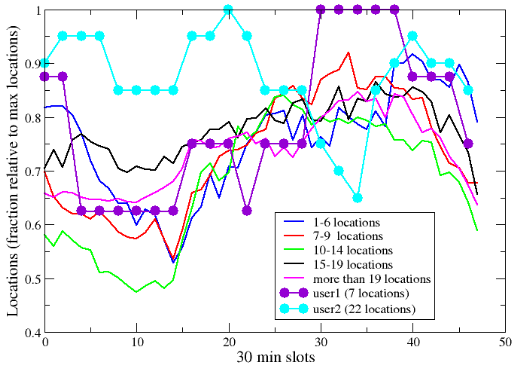


**Supplementary Figure** 11: Number of visited locations at different time-slots.

#### 1.3.2 Gaps in the location data

As it became evident from the day vector representation, there are quite many gaps in the location data that have to be handled with. One can develop a set of criteria like: if the gap is smaller than some threshold and the location before and after the gap are the same, then the location has been the same during the whole gap — as it is done in Sec. 1.2.3. The gaps in the data before and at different stages of the gap filling procedure for the first place definition (Sec. 1.2.1) is shown in Fig. 12.

A totally different approach is to take a look at the gaps and their distribution itself and try to find out whether or not any interesting patterns can be

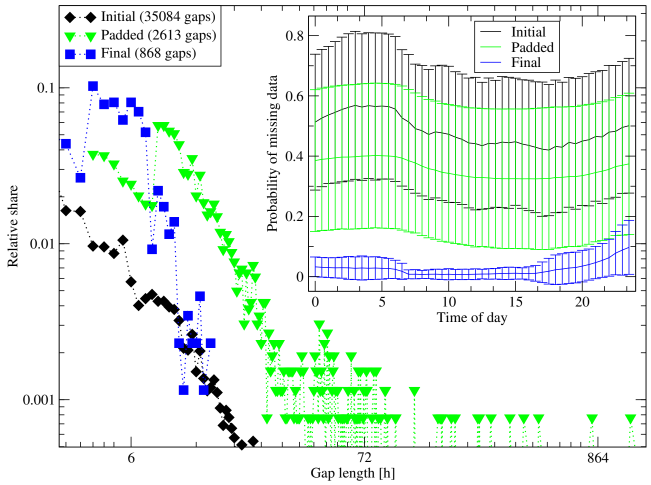


**Supplementary Figure** 12: Large figure: Gap length distribution, Inset: Probability of missing data as a function of time of day. Averages over all accepted users. The resolution is 0.5 h.

found. There is a lot of uncertainty in the following results since the origin and accuracy of the gap and their lengths respectively can not be addressed directly.

The average gap lengths for the accepted users are plotted in Fig. 13, upper panel. No correlation was found between the number of days and average gap length (not shown), but one could try to search for correlation with other aspects of the available data. It is also interesting to note when there is missing data. The probability of missing data is shown in Fig. 13 averaged over all users. First of all the most gaps are found during night time (people turn their phones off during the night ...) and the phones are most likely turned on in the afternoon. However, there are some differences in the gap data between the users. For one user one finds for example two local minima (blue line in Fig. 13) in the gap data at around 10am and 3pm. A possible explanation could for example be group meetings during the work day, where one might turns off the phone.

Further insight can be gained by investigating the distribution of the gap lengths. This can be in a rough sense understood as a distribution of waiting times. It is however not possible to give a single meaning to these waiting times, since these might refer to various topics like: The time after which an empty phone is charged (if the battery was low) The time before the phone is needed again (if it was switched off) The length of an event during which the phone has to be turned off.

The distributions of gap lengths are shown in Fig. 14. What can be noticed from these is that very long gaps (off the order of days) are rare and for gap lengths below 24 h the distributions could be fitted with a power law. However there is not that much available data for a single user and thus the focus is turned to the joint distribution (Fig. 14, lower panel). It is however difficult to figure out how this exponent $\nu$ could characterize a user in a concrete way.

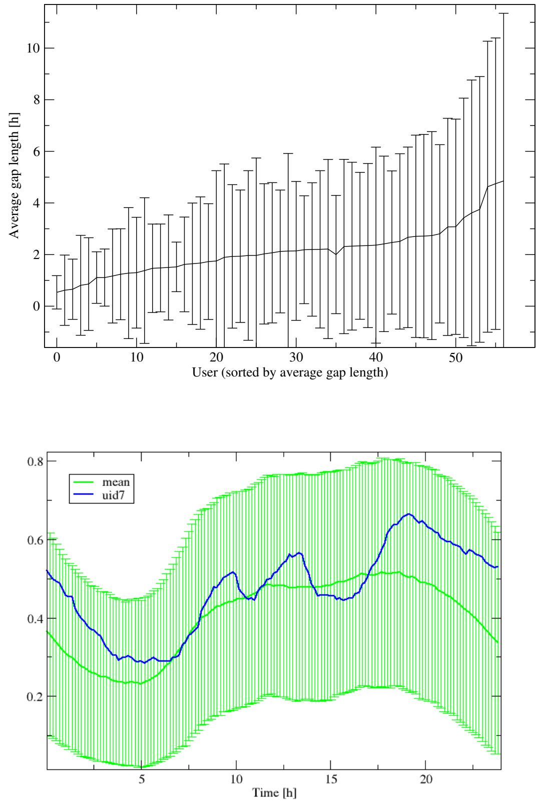


**Supplementary Figure** 13: Upper panel: average gap length for each user (the error bars denote the standard deviation). Lower panel: Gap data at 10 minutes time intervals. Y-axis is the probability that there is location data (no gap) for that time slot. Green line: mean over all users average gap data (the error bars denote the standard deviation). Blue line: average gap data of a single user.

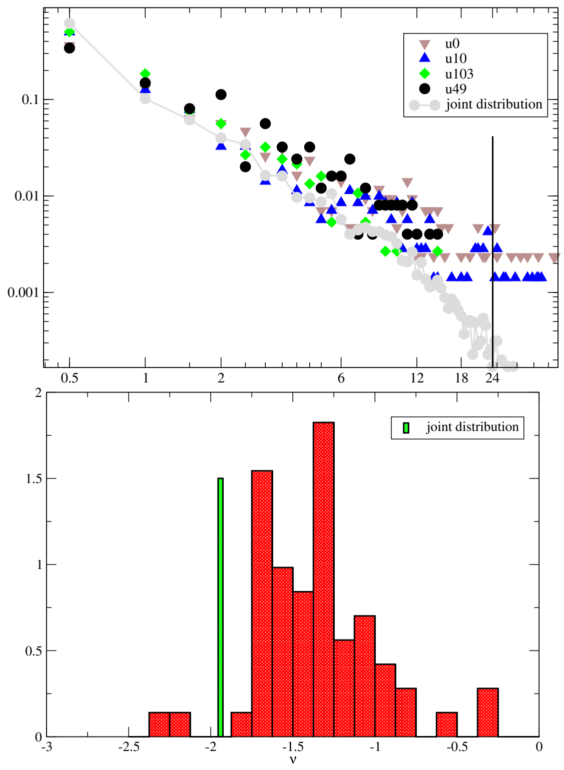


**Supplementary Figure** 14: Upper panel: gap length distribution for a few users and joint distribution of all users. Each color is a different user. Minimum gap size is 30 minutes. Lower panel: Distribution of $\nu$ for gaps between 30 minutes and 24 hours. The resolution is 30 minutes.

Table 3: The $Q$ and cluster number with different time windows for the second user (The second definition of place).

| time window | Q | cluster number |
|---|---|---|
| 0.5 hour | 0.219253 | 58 |
| 1 hour | 0.267696 | 34 |
| 1.5 hour | 0.328684 | 17 |
| 2 hours | 0.351665 | 3 |
| 3 hours | 0.446333 | 6 |
| 4 hours | 0.43417 | 3 |

# 2 Supplementary discussion

## 2.1 Clustering results

The clustering result is affected by the technique applied. Currently we use two different methods.y The first option is to use kmeans clustering. We use hamming distance to measure the difference between two day vectors,

$$d_H(D_1, D_2) = \frac{1}{n}\sum_{j=1}^{n}(1 - \delta(l_{1,j}, l_{2,j})), \tag{2}$$

where $\delta(x, y)$ is the Kronecker delta.

The second method is to create a weighted network for each user. The vertices in the network are the accepted days for that user. The edges are calculated by a weighting function based on the hamming distance,

$$w_{ij} = \exp(-d_H(i, j)\ \beta), \tag{3}$$

where $\beta$ is an adjustable parameter — influencing the slope of the function. Days that share the same *dayvector* have a weight of one and days that have very different *dayvector* respectively a weight close to zero.

The reason to create a weighted network is that one can then apply community detection methods on the network. A remarkable feature of this is that the number of the clusters is in itself an outcome of the algorithm and does not have to be adjusted a priori.

In particular, we use the algorithm proposed by Duch and Arenas [9], which is based on an extremal optimization of the value of modularity and is feasible for the accurate identification of community structure in large complex networks.

In this algorithm, both the length of time window and the weight parameter $\beta$ affect the cluster result. One can actually search for the best time window and $\beta$ of observation for a particular user by optimizing the resulting community structure. In the simplest case one just calculates the modularity with different time windows and hopes for a nice behavior with a clear global maximum. One can then test how much the optimal time window varies between the users. The obtained results are of course also dependent on how accurate the actual place definition is. Tab. 3 presents $Q$ and the number of clusters on different time window.

Irrespective of the used method for clustering, a set of clustered days is achieved for a user. Next one should try to gain as much information as possible from these. When the previous steps have all been successful, which is what is assumed here, a cluster can be interpreted as representing an average day

of a user including fluctuations around it. The number of clusters can thus be directly seen as the number of average days a user has (or more precisely: had during the time of observation) — if it is an outcome of the clustering procedure. Other observables of interest are of course the size of the cluster and whether or not there is any pattern for the days belonging into a specific cluster (e.g. is there a "weekend cluster").

The average day of a cluster can easily be defined as

$$D_{avrg,c} = \{i_{\max,1c}, i_{\max,2c}, ...., i_{\max,nc}\}, \tag{4}$$

where $i_{\max,jc}$ is such that

$$l_{i_{\max,jc}jc} = \max_i(l_{ijc}) \tag{5}$$

and $l_{ijc}$ is the number of times place $i$ is the most dominant place at timeslot $j$ in cluster $c$. Further we allow missing data in the average day only if it is the only place for a given timeslot in the cluster.

A general result is that while many clusters are of reasonable size and can be categorized quite effectively, there are also clusters where this is not the case. This is most notably true for the smaller clusters that do not necessary represent any typical pattern of a users life but are often due to an unique event — for example a trip. Among the dataset were also users who spent most of there time in one place only, probably their home and work place were so close, that these were not detected as separated places by the method used. For these users the clustering method does not provide much of useful information.

### 2.1.1 Entropy after clustering

Besides identifying the behavior a cluster is representative for, it is also relevant to know how much fluctuations there is inside a cluster. In other words one wants to know how regular the days are within a single cluster for one user. One also wants to know how much information is gained by clustering the days and how much uncertainty is still left. Next we restate the definitions of entropy for completeness.

The method of choice in this work is to use an entropy based analysis. One can calculate an entropy for the unclustered time-slot representation for each user by

$$\varepsilon = -\frac{\sum_{j=1}^{N} \sum_{i \in I} l_{ij}/D_{tot} \ log(l_{ij}/D_{tot})}{N}, \tag{6}$$

where $N$ is the number of time slots in one day (columns), $D_{tot}$ is the total number of days (rows), $I$ is the set of all places the user has visited and $l_{ij}$ is the number of times place $i$ is the most dominant place at timeslot $j$. Since missing data is no real place, it is here not included in $I$. A low entropy means that the days of a user are regular and vice versa. The number of accepted locations does affect the unclustered entropy — this is shown in Fig. 15.

In a similar manner one can calculate the entropy for a cluster of days as

$$\varepsilon_c = -\frac{\sum_{j=1}^{N} \sum_{i \in I} \ l_{ijc}/D_c \ log(l_{ijc}/D_c)}{N} \tag{7}$$

where $D_c$ is the number of days that belong to cluster $c$ and $l_{ijc}$ is the number of times place $i$ is the most dominant place at timeslot $j$ in cluster $c$.

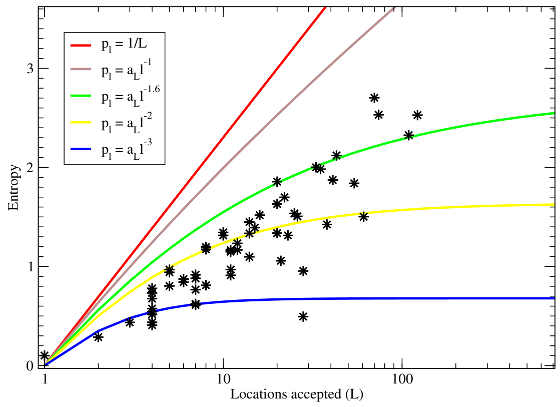


**Supplementary Figure** 15: Unclustered entropy as a function of locations (accepted) with place definition def1. Also shown in the figure is the unclustered entropy that would be obtained if the locations would by evenly distributed and for power-law distributions with different exponents. No power-law distribution does fit the data really well, particularly the exponent for the histogram of the significant locations (e.g. Fig. 1 of the manuscript) overestimates the unclustered entropy for most users.

The entropy related to the clustered days should be smaller than for the unclustered case. The clustered entropy is here defined as the weighted average of the single cluster entropies:

$$\bar{\varepsilon}_c = -\frac{\sum_{c\in C}\sum_{j=1}^{N}\sum_{i\in I} l_{ijc}\ log(p_{ijc})}{D_{tot}N} \tag{8}$$

where $p_{ijc} = l_{ijc}\ /D_c$ and $C$ is the set of clusters. The drop in entropy due to the clustering can further be defined as

$$\Delta\varepsilon_{[\%]} = 100\ (1 - \frac{\bar{\varepsilon}_c}{\varepsilon}). \tag{9}$$

When comparing two clusterings for the same *dayvectors*, the better one will result in a higher $\Delta\varepsilon[\%]$.

The percentual entropy drop for different clustering methods are shown in Fig. 16 for place definition one and in Fig. 17 for place definition two. From these figures one can observe that EO performs on average better the higher the unclustered entropy — this is most likely due to the fact that the number of clusters is larger than the prefixed value for kmeans. One should notice here that the clustered entropy does not employ any kind of cost for the number of clusters. Thus a situation where each day forms its own cluster would result in a clustered entropy of zero. Strictly speaking the clustered entropy can not be compared independently but should be put into prospective with the amount of clusters. The entropy drop as a function of clusters found is shown in Fig. 18 for place definition one and in Fig. 19 (A) for place definition two respectively.

In Fig. 15 it was clearly seen that the number of locations does influence the unclustered entropy. It is however not directly clear if it does also so for the entropy drop. From Fig. 20 and Fig. 21 , where the two quantities are compared for place definition one and two respectively, no clear correlation between the number of locations and the entropy drop was found except for a small decrease in the entropy drop with more locations. Intuitively one might argue that on

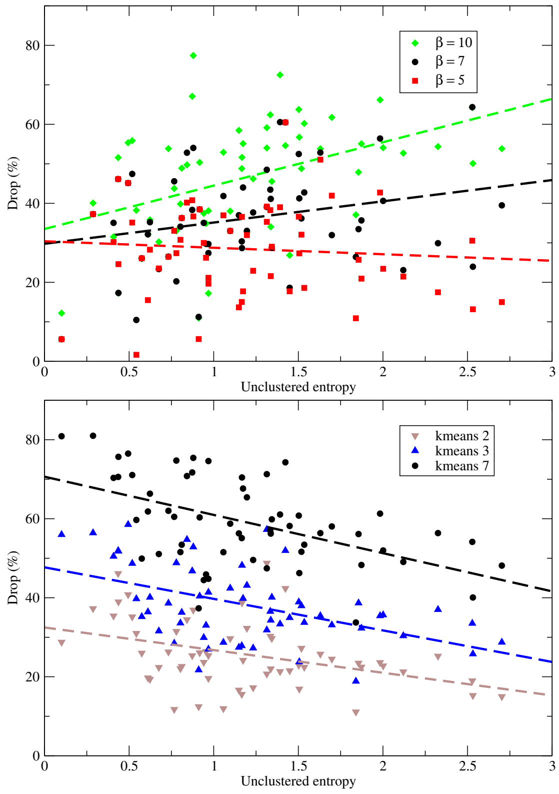


**Supplementary Figure** 16: Percentual drop in entropy as a function of unclustered entropy for different clustering methods

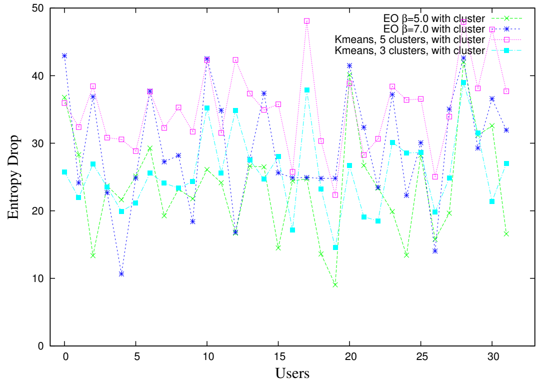


**Supplementary Figure** 17: Percentual entropy drop due to clustering

average for a user with more important locations there are on average also more typical days ergo clusters. A slight trend of this kind is indeed seen in Fig. 22 for the first place definition - here a logarithmic fit seems to work well. However in Fig. 23 with the second place definition no dependency of this kind is detectable unambiguously. Thus one might argue that the observed log scaling is mostly an artifact due to the rule to set the number of locations or that for the second place definition the number of locations is too small, or otherwise truncated in order to obtain a clear dependency. In any case the observed scaling is not

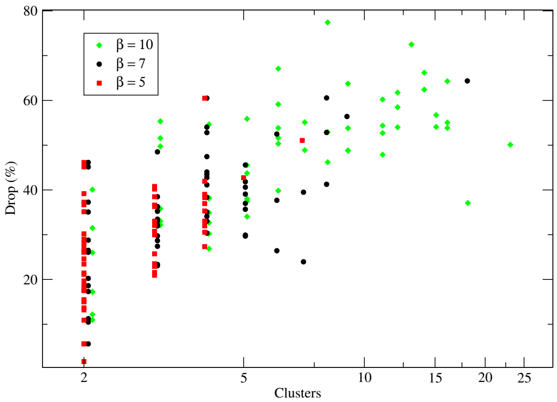


**Supplementary Figure** 18: Entropy drop (place definition one) as a function of clusters found.

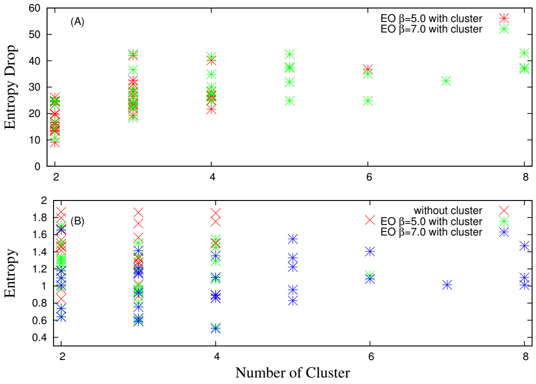


**Supplementary Figure** 19: (place definition two) A) Percentual entropy drop due to clustering as a function of the number of clusters. B) Entropy as a function of the number of clusters.

linear. Another attempt would be to try to relate the entropies to the user-to-user variation resulting from the number of significant locations per user. To this end, we show in Figure 24 the cumulative distributions of entropy for tw cases: clustered, bare, and the distribution of the logarithm of the mixing entropy ($N-1$, where $N$ is the number of locations per user). The data has been rescaled with the median of the quantity in each case. As noted in the main text the two entropy distributions are quite close; the location data differs in the tails. This happens in an intuitively transparent manner: the distribution is more narrow than for the entropy.

It is clear that most humans have life patterns that repeat from day to day. Thus it is also of interest to take a look at the entropy of a user at the scale of the single time slots. The entropy for a single timeslot is in analogy to the previous definitions obtained by

$$\varepsilon(j) = -\sum_{i \in I} l_{ij}/D_{tot}\ log(l_{ij}/D_{tot}) \tag{10}$$

where $j$ is the timeslot, $D_{tot}$ is the total number of days (rows) in the cluster,

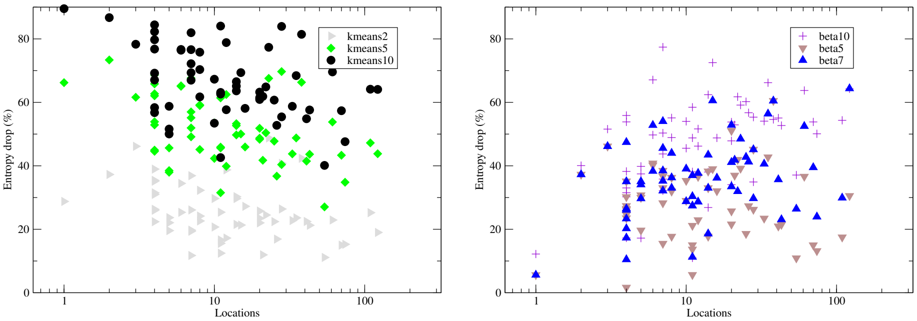


**Supplementary Figure** 20: (place definition one) Percentual drop in entropy as a function of accepted locations for different clustering methods.

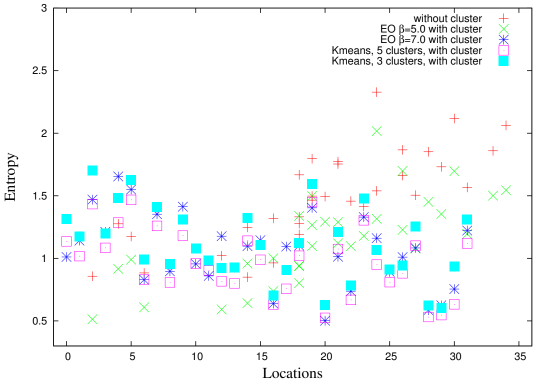


**Supplementary Figure** 21: (place definition two) Unclustered entropy with the number of locations.

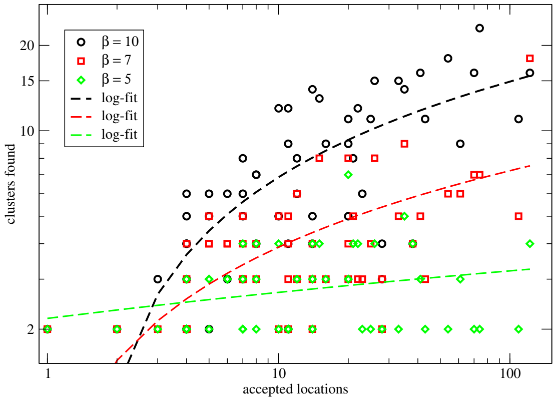


**Supplementary Figure** 22: (place definition one) Number of clusters found with the number of locations.

$I$ is the set of all places the user has visited in this cluster and $l_{ij}$ is the number of times place $i$ is the most dominant place at timeslot $j$. A higher entropy at a certain timeslot indicates that the user is more likely to deviate from the average behavior at that time. A entropy of zero is obtained only if the user is always at the average place for that timeslot and cluster. A few examples are

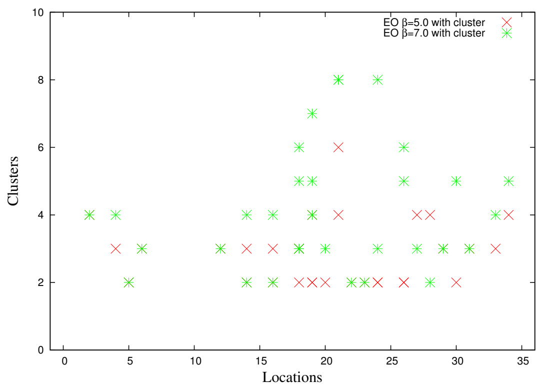


**Supplementary Figure** 23: (place definition two) Number of clusters found with the number of locations.

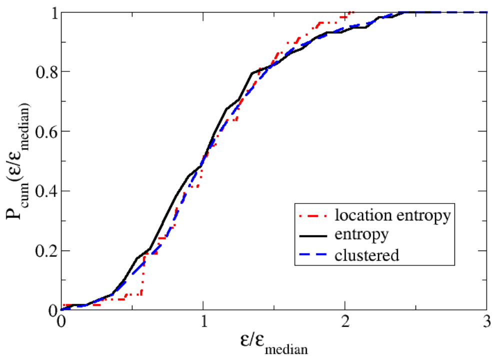


**Supplementary Figure** 24: Entropy and locations (per user) cumulative distributions scaled with the median values.

shown in Fig. 25.

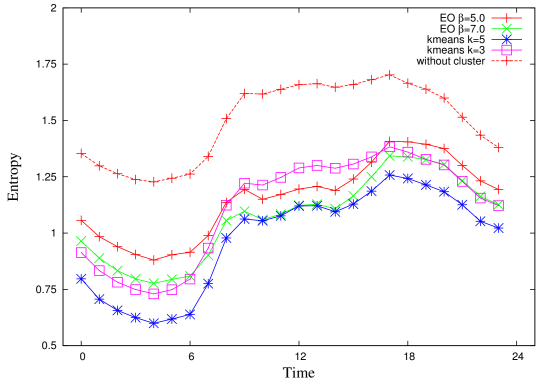


**Supplementary Figure** 25: (place definition two) Average entropies with time of day under different cluster algorithm.

The final issue that we must answer is: given the power-law distribution

of the time spent at various locations (Fig. 10), how is it actually feasible to have a relatively limited number of patterns for all users and how does that relate to the entropy? A location can be found in the data at a slot with four different roles: i) it is the right one in the pattern, ii) it is in the pattern but at another slot(s), iii), it is in another pattern and iv) it is not actually part of *any pattern*. The case ii) relates to eg. occurrences when the working day length at hand varies from the one predicted by the pattern (see below for this issue). We distinguish between cases iii) and iv) and plot in Figure 26 the fraction of the occurrences of a location such that it is "out of patterns". This is true noise - measured partly by the entropy - coming from the presence of rare activities/locations in the data. What is quite intriguing is that below a typical frequency, the probability for a location to be "pure noise" as measured by in case iv) increases as a power-law. The reason for this functional relation is as unclear as that of the power-law for the location frequencies, but the message is simple and not unexpected: rare activities are not found in typical patterns. Note also the implication this might have for the relation between entropy and the number of important locations for each person.

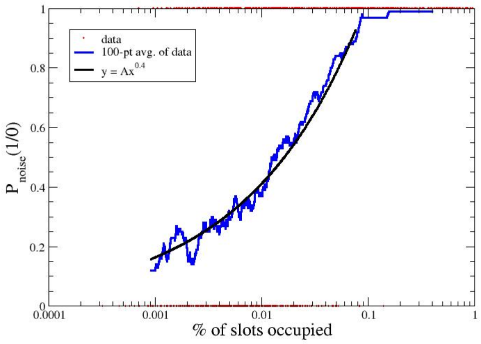


**Supplementary Figure** 26: Fraction of "clustered" locations according to their prevalence in the location data.

## 2.2 Predictability

Our discussion is based on the concept of a "meaningful location". This is a coarse-grained description that avoids following accurately the real-time geographic position. As the radius of such locations is smaller than 1 km, any predictions of users' behavior on this level could also be used in many applications, like traffic prediction. We note that further improvements can be done in the prediction accuracy by changing or augmenting the location data (Bluetooth, WLAN, GPS...). The mobile phone base station methods have intrinsic limitations due to the varying density of stations, and due to the technology limitations discussed in data analysis part.

Just like weather forecasting gives us a probability of precipitation, we also define the probability of user's (next) slot location as the prediction. When we try to form such a guess, usually we cannot say with certainty that the user will goto place A at next change of slot (eg. by the hour), but a probabilistic description like user will go to $A_1$ with probability $a_1$%, and to $A_2$ with

probability $a_2$%... Thus for user $i$, in time window $t$, we define the quality of prediction as,

$$\Pi_{i,t} = \overline{\Pi}_{A(i,t)}, \tag{11}$$

where the $A(i,t)$ is the event that the user $i$ at some place at time $t$ and the $\Pi_{A(i,t)}$ is the prediction probability we give for this event. Therefore, the overall prediction quality of user $i$ can be figured out by computing the average of $\Pi_{i,t}$ with $t$, and the overall prediction quality of time window $t$ can be given by the average with $i$.

As we discussed above the cluster structure decreases the entropy, and it also helps us to improve the accuracy of prediction. Over what we can predict without the knowledge of actual cluster. In the following, we will discuss the prediction accuracy in both cases. As in the case of entropy, we also apply two methods to predict users' behavior. The simplest one is to predict the slot locations by expected main one. The other one uses the empirical knowledge of the transition matrix from location $A_1(i,t)$ to $A_2(i,t+1)$. As is obvious, the latter method uses more detailed information and can thus be expected to give more reliable results.

### 2.2.1 Method 1: The expectation of daily life.

The first prediction method comes from the measurement of the "center of gravity" of the user's daily life in each time slot as we measure the probability of a user at all possible places at a certain time window from the data set. Then we use this probability as the actual prediction; the resulting inaccuracy or error is obviously related to the entropy in general and the entropy for the given time of the day per user, in particular. When we consider the cluster, we define the $p_{iatc}$ as the probability of a user $i$ at place $a$ at time window $t$ in cluster $c$. Therefore, the quality of prediction $\Pi_{i,t}$ can be written as

$$\Pi_{i,t} = \overline{\sum_a p_{iatc}^2}, \tag{12}$$

where the average is over all clusters. In the absence of cluster, we just need to use $p_{iat}$ instead of $p_{iatc}$.

The prediction accuracy at different time windows is shown in Fig. 27, upper panel. We can see that the prediction quality improves clearly with clustering. The average prediction accuracy is 0.528 (with clustering) and 0.361 (without clustering). Note the strong effect of the clustering method on the prediction. It is also interesting to note the daily variation in the prediction quality, which shows similar features for all the three cases depicted.

### 2.2.2 Method 2: Transition matrix

The transition matrix $T_{t,t'}$ contains the probabilities of where the user is at time $t'$ when the user at some place at time $t$ and with $t' = t + \Delta t$. If the user stays at $A_1$ at time slot $t$, the probability of user moving to $A_2$ is $T_{t,t+1}(A_2|A_1)$. So the $A_1$th row of the resulting matrix is the resulting prediction. The prediction accuracy at time slot $t$ is,

$$\Pi_t = \overline{\sum_{A(t),A(t')} T_{t,t'}(A(t)|A(t'))^2}, \tag{13}$$

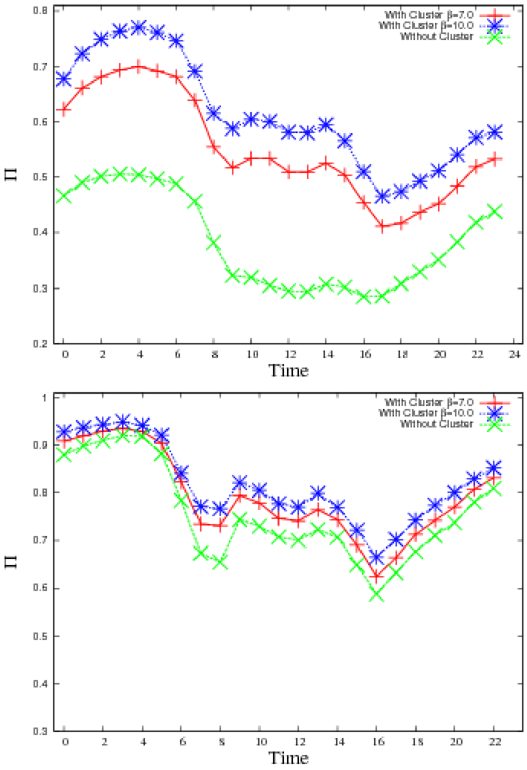


**Supplementary Figure** 27: Upper panel: (Place definition two) The average prediction quality of all available users by the first method in the cases of using clusters and without. The clustering is from the EO algorithm with $\beta$=7.0 and $\beta$=10.0. Lower panel: the same for the second place definition method.

where $A(t)$, $A(t')$ are the user's locations at time slots $t$ and $t'$.

Figure 27, lower panel presents the prediction accuracy with the time lag $\Delta t = 1$. Because we use the user's location in the past in this prediction method, we can see that the prediction accuracy is much higher than the first one and the clustering cannot help to improve the accuracy a lot. The average prediction accuracy is 0.7412 (without clustering) and 0.7796 (with clustering). The implication is to repeat that the matrix $T$ contains indirectly the information revealed through the clustering: certain elements are non-zero since they correspond to location changes in a day whose structure is described by one of clusters, and the remaining ones are so, since the clustering is nevertheless noisy.

We can see in both cases/methods, that the prediction quality is better at night than day. The obvious significance is that is because the user will sleep at home at night and they will visit a lot of places during the daytime. The prediction quality has two minima around 9:00 and 17:00 when users go to work and go back home on the workday. In the workday morning, the users will go to nowhere but their offices, but when they leave the office, they have more choices like home, shopping, gym, bar, and so on. Therefore the latter minimum around 17:00 is the worst prediction time window.

The predictions can be extended also to $\Delta t > 1$. The user location predictions over a longer range or the prediction accuracy with different $\delta t$ and different clustering parameters are depicted in Fig. 28. Two observations stand

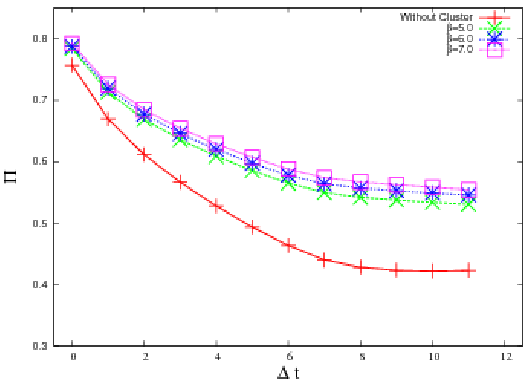


**Supplementary Figure** 28: Place definition two) The average prediction quality of all available users by the second method for $\Delta t > 1$ with different clustering parameters $\beta$.

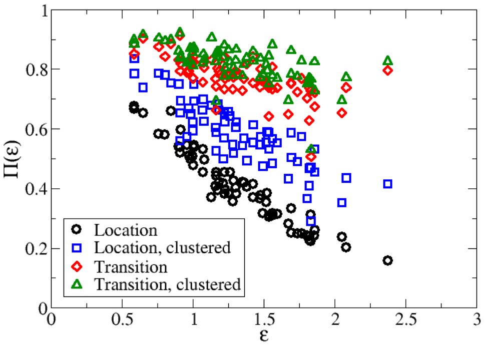


**Supplementary Figure** 29: (Place definition two) The users' raw entropy against the prediction accuracy from both the methods and with and without clustering. The clustering is from the EO algorithm with $\beta$=7.0.

out. First of all, the long-range prediction with the transition matrix technique decays fairly slowly - recall the time slot length here is 1 hour so the range of time delays in the figure extends to 12 hours. Even at such times the achieveable prediction accuracy remains comparable to that of the first method. This is of course natural in that the null case is "I go at N slots from now to the most likely slot at time N+1". Second, for larger delays $\Delta t > 1$ the clustering improves the accuracy, and the longest delays/lags still give a slightly better result than the first method with clustering.

We know a small entropy indicates a regular life which should be easier to predict. Therefore, we would like to see if we can predict a user's behavior better when he/she has a smaller entropy. Figure 29 presents the prediction accuracy with the entropy of 66 users. It is interesting to compare the user-to-user variability given the original entropy and the influence of the particular prediction methods and clustering. The figure is another variant of Figure 4c of the main manuscript.

Figure 30 discusses the matter further by turning to the cumulative his-

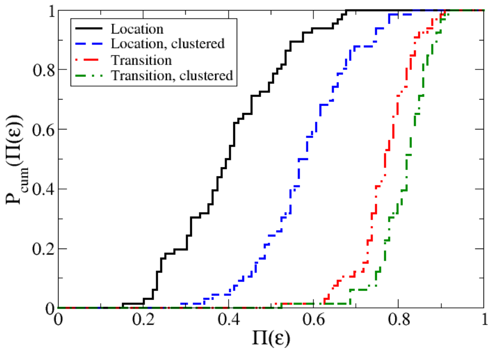


**Supplementary Figure** 30: (Place definition two) The cumulative distributions of the prediction levels for the four different cases (two methods, with or without patterns) as above.

tograms of the methods. In all four prediction cases there are indications that a small fraction, less than 10 % of the user population studied here, are difficult to predict. The second, transfer matrix method, shows a clear but quantitatively smaller improvement 0.0384 by the use of patterns, whereas in the first method there is a typical improvement of about 0.167 in the prediction efficiency. The maximum predictabilities indicated by the histograms vary from about 2/3 (location, or first method) to about 0.82 (adding patterns) to 0.91 - 0.92 (second method). It is relatively obvious that for very regular persons the transfer matrix idea works equally well with and without the utilization of underlyign patterns of life.

## 2.3 Voice call and Message data: Work-Home cycle

In addition to predictability based on location data, we also tried to gain further insight by considering the individual communication patterns. Since the data allows to identify individual events (time of communication), type (call, text message, out/inbound), and "persons" (distinct communication partners), it seems an appealing idea that correlations might exist between *deviations* from the typical pattern and such events. In short, the idea was to predict the departure from work to the next "location".

Three different approaches were tried. We explored the possibility of fluctuations in communication densities ("more calls and/or messages in the last three hours before leaving. In particular, the possibility of "bursts" in the activity were looked-for, in cases when the average working-day length was shorter than the average for the current user. The data indicated no statistically significant signatures of such, however. The second approach was to look for a "typical" event in terms of the more frequent numbers found from the total communication statistics. This also did not lead to any measurable correlations: the idea would have been that there are regular deviations arising from daily-life needs that compete with the work requirements. Thus the right type of a call or a message would induce a change in the planned daily activities and a departure from work. Finally, we also considered the "anti-correlations" or the effect of communications with rarely found numbers in the data. Here the idea would

have been that in the minority of cases, the working day becomes shorter than usual since something unusual happens. This would have then been correlated with the presence of a communication with a "strange phone number". Also this try lead to no significant results. It is worth noting that the typical communication density from the data during say the last two hours prior to departure from work is 0-2 calls and messages per half an hour time slot, so in a way it is not surprising that for a typical user it is difficult to establish even a weak correlation of any particular kind, since the smallest possible fluctuation is one call/sms per time slot.

The lack of evident correlations then leads to the question, if the duration data of the working day has some particular characteristics and why. We found that of the total users contained in the statistics (58 users) 30 or 52 per cent, a slight majority, seemed to have enough similarity to assume that they each follow a similar probability distribution function for the working day length, albeit with different, "personal" parameters for each. Of the remaining users at least some are described by a non-sufficient quantify of data. Figure 31 shows the individual parameters for the empirical probability distributions for each user, for both those users chosen for the modeling attempt described below and for the others. We notice that the work-day length is smaller for the second set, but it is crucial to note that this claim is based on the *significant location*, ie. the idea that the work-day is based on one single (though possibly "diffuse" or locally spread) geographic location. The standard deviations are comparable, while the skewnesses are clearly different and have even different signs. Also, the average number of days in the statistics is about three times smaller for the "deviant" cases.

To analyze the data and develop a toy model, we formed an aggregate distribution by rescaling and summing. Thus, all individual probability distributions were scaled to a zero mean and a variance of unity, and then summed together. Figure 32 shows the resulting data; it is obvious that the tail versus smaller-than-average working day lengths is broader.

The red curve shows one fit of the following simple model (the fit can be done in various ad hoc ways depending on whether one ignores the central peak or not). Consider that the working day length is completely deterministic in that a person expects to work X hours and Z minutes a day. Valid examples would be the case in which bus timetables or employer-stipulated expectations set the norm. Deviations occur due to leaving earlier, or since the current-day's tasks need to be finished before leaving. The model that we use to fit the data is based on two processes: if I need to leave from work for any pertinent reason before my usual (and known) average, the chance for this increases by every moment that passes. Likewise, the same idea but with a different parameter applies to staying at work longer: the likelihood of staying for the next $\Delta t$ decreases by a constant amount at each "step" or time I decide. There are thus two exponential tails, which follow from two different biases (to stay or to go) and the deterministic expectation of a typical working day. The exponential scale parameters are $t_1 = 80.8917(minutes)$ and $t_2 = 40.9643(minutes)$, respectively, for the two tails.

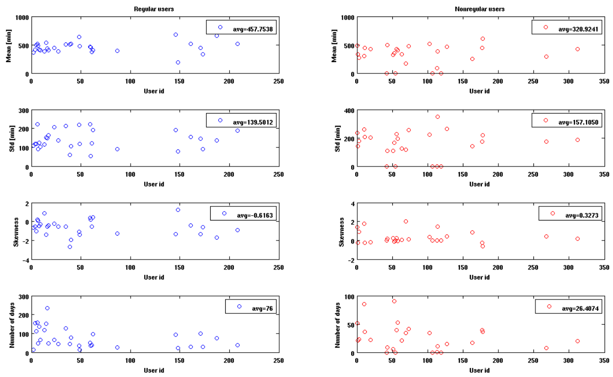


**Supplementary Figure** 31: Key statistics of regular (left) and irregular users (right) as a function of user id.

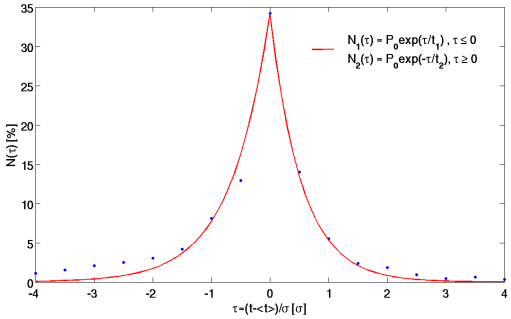


**Supplementary Figure** 32: Length-of-working-day distribution and the model fit for regular users.

# References


[1] M.R.L. Hodges, The GSM radio interface, British Telecom Technology Journal, 8(1): 31-34 (1990).

[2] E. Trevisani and A. Vitaletti, Cell-ID location technique, limits and benefits: an experimental study, Proceedings of the Sixth IEEE Workshop on Mobile Computing Systems and Applications: 51-60 (2005).

[3] K. Laasonen, Mining Cell Transition data, Ph.D. dissertation, University of Helsinki (2009).

[4] P. Nurmi, Identifying Meaningful Places, Ph.D. dissertation, University of Helsinki (2009).

[5] U. Ahmad, B. J. d'Auriol, Y. Lee and S. Lee, The election algorithm for semantically meaningful location-awareness, Proceedings of the 6th international conference on Mobile and ubiquitous multimedia: 55-63 (2007).

[6] D. H. Kim, J. Hightower, R. Govindan and D. Estrin, Discovering semantically meaningful places from pervasive RF-beacons, Proceedings of the 11th international conference on Ubiquitous Computing: 21-30 (2009).

[7] N. Eagle and A. S. Pentland, Eigenbehaviors: Identifying structure in routine, Behavioral Ecology and Sociobiology, 63(7): 1057-1066 (2009).

[8] M. E. J. Newman and M. Girvan, Finding and evaluating community structure in networks, Phys. Rev. E 69: 026113 (2004).

[9] J. Duch and A. Arenas, Community detection in complex networks using extremal optimization, Phys. Rev. E 72: 027104 (2005).
